# Compositional and physicochemical factors governing the viability of *Lactobacillus rhamnosus* GG embedded in starch-protein based edible films

**DOI:** 10.1016/j.foodhyd.2015.08.025

**Published:** 2016-01

**Authors:** Christos Soukoulis, Poonam Singh, William Macnaughtan, Christopher Parmenter, Ian D. Fisk

**Affiliations:** a Division of Food Sciences, School of Biosciences, University of Nottingham, Sutton Bonington Campus, Loughborough LE12 5RD, Leicestershire, United Kingdom; b Nottingham Nanotechnology and Nanoscience Centre, University of Nottingham, University Park, Nottingham NG7 2RD, United Kingdom; c Environmental Research and Innovation (ERIN) Department, Luxembourg Institute of Science and Technology (LIST), 41 Rue du Brill, L-4422 Belvaux, Luxembourg

**Keywords:** Probiotics, Rice starch, Corn starch, Gelatine, Sodium caseinate, Soy protein

## Abstract

Probiotic incorporation in edible films and coatings has been shown recently to be an efficient strategy for the delivery of probiotics in foods. In the present work, the impact of the compositional, physicochemical and structural properties of binary starch-protein edible films on *Lactobacillus rhamnosus* GG viability and stability was evaluated. Native rice and corn starch, as well as bovine skin gelatine, sodium caseinate and soy protein concentrate were used for the fabrication of the probiotic edible films. Starch and protein type both impacted the structural, mechanical, optical and thermal properties of the films, and the process loss of *L. rhamnosus* GG during evaporation-dehydration was significantly lower in the presence of proteins (0.91–1.07 log CFU/g) compared to solely starch based systems (1.71 log CFU/g). A synergistic action between rice starch and proteins was detected when monitoring the viability of *L. rhamnosus* GG over four weeks at fridge and room temperature conditions. In particular, a 3- to 7-fold increase in the viability of *L. rhamnosus* GG was observed in the presence of proteins, with sodium caseinate – rice starch based films offering the most enhanced stability. The film's shelf-life (as calculated using the FAO/WHO (2011) basis of 6 log viable CFU/g) ranged between 27-96 and 15–24 days for systems stored at fridge or room temperature conditions respectively.

## Highlights

• Edible hydrogels (rice or corn starch) were formed containing *Lactobacillus rhamnosus*.
• Protein inclusion improved stability during drying and shelf life storage.
• Rice based films were more stable than corn based films (4 °C).
• Rice based films were more compact and less elastic than corn based films.
• Sodium caseinate inclusion improved shelf life stability.

## Graphical abstract

**Figure:**
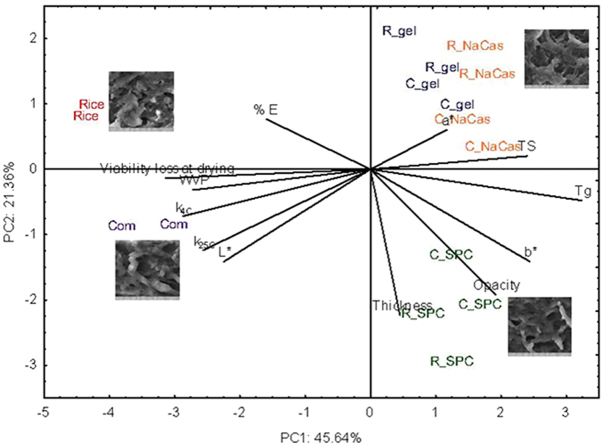

## 1 Introduction

The term probiotics refers to live organisms, which when administered in adequate amounts (FAO/WHO, 2011), confer a health benefit on the host (FAO/WHO, 2002). Probiotics exert a broad spectrum of beneficial health effects including reduction of the relapse frequency of *Clostridium dificile* or Rotavirus associated diarrhoea, reduction in the symptoms of irritable bowel syndrome and inflammatory bowel disease, modulation of the immune system, reduction of lactose intolerance symptoms and prevention of atopic allergies (Saad, Delattre, Urdaci, Schmitter, & Bressollier, 2013). Delivery of sufficient viable cells can be quite restrictive for food manufacturers as a considerable amount of living cells are inactivated during food processing (heat, mechanical and osmotic stress), storage (exposure to acute toxic factors such as oxygen, hydrogen peroxide and water vapour) or during interaction with the matrix (Jankovic, Sybesma, Phothirath, Ananta, & Mercenier, 2010). In addition, disintegration and passage of the ingested food matrix through the gastrointestinal tract can also critically impact the colonisation ability and the composition of the probiotic intestinal microbiota (Cook, Tzortzis, Charalampopoulos, & Khutoryanskiy, 2012).

Encapsulation is a physicochemical or mechanical process that has been successfully implemented to retain cell viability under sub-lethal environmental conditions. It can also be used to delay release of the encapsulated living cells during gastro-intestinal transit (Burgain, Gaiani, Linder, & Scher, 2011; Cook et al., 2012). To date technologies based on cell entrapment in dehydrated matrices (using spray, freeze or fluidised bed drying) and cross-linked biopolymer based micro-beads are the most common routes to maintain probiotic efficacy (Burgain et al., 2011; Soukoulis, Behboudi-Jobbehdar, Yonekura, Parmenter, & Fisk, 2014b; Soukoulis et al., 2014). Immobilisation of living cells either by physical entrapment in biopolymer networks (e.g. cross-linked or entangled polysaccharide hydrogel systems) or by absorption/attachment in pre-formed carriers and membranes is a well-established strategy for microbial stability in other industries. Examples include biomass production (lactic acid and probiotic starters), fermentation (wine, milk) and metabolite production such as lactic, citric acid, bacteriocins and exopolysaccharides (Kourkoutas, Bekatorou, Banat, Marchant, & Koutinas, 2004). In addition, immobilisation of probiotic bacteria in edible films or coatings has been recently introduced as a novel method for the encapsulation of probiotics (Altamirano-Fortoul, Moreno-Terrazas, Quezada-Gallo, & Rosell, 2012; Kanmani & Lim, 2013; López de Lacey, López-Caballero, & Montero, 2014; Soukoulis et al., 2014b). López de Lacey, López-Caballero, Gómez-Estaca, Gómez-Guillén, and Montero (2012) reported that *Lactobacillus acidophilus* and *Bifidobacterium bifidum* entrapped in gelatine based coatings stored for 10 days at 2 °C showed extended shelf life and prolonged viability. In their study, Kanmani and Lim (2013) reported that the viability of multiple probiotic strains e.g. *L. reuter*i ATCC 55730, L. plantarum GG ATCC 53103 and L. acidophilus DSM 20079 in starch-pullulan based edible films was strongly influenced by the pullulan to starch ratio and storage temperature. Similarly, in a series of studies we have found that the viability of *Lactobacillus rhamnosus* GG in edible films is strictly dependent on the composition of the matrix, with whey proteins and prebiotic soluble fibres promoting the stability of *L. rhamnosus* GG during air drying (37 °C for 15 h) and storage (4 and 25  °C at 54% RH) (Soukoulis et al., 2014; Soukoulis et al., 2014b). We have also demonstrated the feasibility of polysaccharides - whey protein concentrate based edible films as effective carriers of probiotics in pan bread (Soukoulis et al., 2014). The coating of bread crusts with a probiotic containing film enabled the production of probiotic bakery products which can deliver live probiotic cells under simulated gastrointestinal conditions without any major changes to the physicochemical, texture or appearance of bread (Soukoulis et al., 2014).

The aim of the present work was to investigate the impact of the compositional, physicochemical and structural properties of binary starch-protein edible films on *L. rhamnosus* GG viability and stability. Binary films were chosen to offer greater processing flexibility to the films and enhance *L. rhamnosus* GG viability and stability. A series of edible films comprising native starch (either rice or corn) and a protein, either sodium caseinate, soy protein concentrate or bovine gelatine type II, were prepared with *L. rhamnosus* GG and subsequently evaluated for their ability to entrap and stabilise *L. rhamnosus* GG. The resulting physical, structural, optical and thermal properties of the probiotic films were characterised.

## 2 Materials and methods

### 2.1 Materials

A *L. rhamnosus* GG strain with established probiotic activity was used (E-96666, VTT Culture collection, Espoo, Finland). Native starch isolated from rice or corn and bovine skin gelatine Type II was obtained from Sigma–Aldrich (Gillingham, UK). Soy protein concentrate (SPC) and sodium caseinate were purchased from Acron Chemicals (Birmingham, UK). Glycerol (purity >99%) was used as plasticising agent (Sigma–Aldrich, Gillingham, UK).

### 2.2 Stock culture preparation and growth conditions of *L. rhamnosus* GG

One mL of sterile phosphate buffer saline pH 7.0 (Dulbecco A PBS, Oxoid Ltd., Basingstoke, UK) was added to the lyophilised culture of *L. rhamnosus* GG and after adequate mixing, the bacterial aliquot was streaked onto MRS-agar medium (MRS Agar, Oxoid Ltd., Basingstoke, UK). The samples were cultured under anaerobic conditions in hermetically sealed plastic containers containing Anaerogen® (Oxoid Ltd., Basingstoke, UK) at 37 °C for 48 h. A small amount of the colonies was collected with a sterilised loop and suspended in the cryo-medium of the Microbank systems (Pro-Lab Diagnostics UK, Merseyside, UK). The plastic bead cultures were stored in a freezer at −80 °C (Behboudi-Jobbehdar, Soukoulis, Yonekura, & Fisk, 2013).

One bead of the deep frozen cultures was placed in MRS broth (Oxoid Ltd., Basingstoke, UK). Aliquots were incubated for 48 h at 37 °C under anaerobic conditions in plastic jars. Cell pellets were collected by centrifugation (3000 g for 5 min). The supernatant was discarded and cells were washed twice using phosphate buffer saline pH 7.0.

### 2.3 Preparation of the film forming solutions

Two individual starch and six binary starch: protein (1:1) film forming solutions containing 4% w/w biopolymer total solids were prepared by dispersing the dry materials (native starch and protein) in distilled water at 50 °C under agitation for 1 h. After the addition of the plasticiser at a level of 30% (i.e. 1.2% w/w) of the total biopolymer solids, the aqueous dispersions were adjusted to pH 7.00 ± 0.05 using sodium hydroxide (0.1 M). Samples were then heated to 90 °C for 20 min to complete starch gelatinisation and protein denaturation and destroy any pathogens. The film forming solutions were then cooled to 40 °C until inoculation with *L. rhamnosus* GG pellets.

### 2.4 Preparation and storage of the edible films

One hundred mL of each film forming solution was inoculated with *L. rhamnosus* GG (6 pellets) and degassed (40 °C for 10 min). Thirty mL of each solution was aseptically transferred to sterile petri dishes (inner diameter 15.6 cm; Sarstedt Ltd., Leicester, UK) and the films were cast (37 °C for 15 h) in a ventilated incubator (Sanyo Ltd., Japan). Dry films were peeled intact and conditioned at room (25 ± 1 °C; ca. 54% RH) or fridge temperature (4 ± 1 °C; ca. 59% RH) in desiccators containing saturated magnesium nitrate solution. Separate films (10 × 10 cm^2^ individual squares, stored and conditioned at 25 °C; 54% RH, 3 d), were made for the characterisation of the physicochemical, mechanical and structural properties of the probiotic edible films.

### 2.5 Enumeration of *L. rhamnosus* GG

One mL of the probiotic film forming solution was suspended in 9 mL of sterile PBS and vortexed for 30 s to ensure adequate mixing. The method described by López de Lacey et al., (2012) with minor modifications was adopted for the recovery of *L. rhamnosus* GG from the bread crust. More specifically, 1 g of edible film containing *L. rhamnosus* GG was transferred to 9 mL of sterile PBS and left to hydrate and dissolve under constant agitation in an orbital incubator at 37 °C for 1 h. The resulting solutions were subjected to serial dilutions in PBS. Each dilution was plated on a de Man, Rogosa and Sharpe (MRS) agar (Oxoid Ltd., Basingstoke, UK) and the plates were stored at 37 °C for 72 h under anaerobic conditions to allow colonies to grow. Enumeration of the bacteria was performed in triplicate, following the standard plating methodology (Champagne, Ross, Saarela, Hansen, & Charalampopoulos, 2011) and the total counts of the viable bacteria were expressed as log colony forming units per gram (log CFU/g).

The survival rate of the bacteria throughout the film forming solution drying process was calculated according to the following equation (1).

(1) $$\%\;\text{viability}=100\times \frac{\text{N}}{{\text{N}}_{\text{0}}}$$

where: N_0_, N represent the number of viable bacteria prior to and after the implemented drying process (Behboudi-Jobbehdar et al., 2013).

*L. rhamnosus* GG inactivation upon storage was expressed as the logarithmic value of the relative viability fraction (log N/N_0_). The viability data was fitted to a first order reaction kinetics model as described by the formula:

(2) $$\text{log}\;{\text{N}}_{\text{t}}=\text{log}\;{\text{N}}_{\text{0}}-\text{kT}\;\text{t}$$

where: N_0_, represents the initial number of the viable bacteria and N_t_ the number of viable bacteria after a specific time of storage (CFU/g), t is the storage time (day), and k_T_ is the inactivation rate constant (log CFU/g*day^−^^1^) at temperature, T.

### 2.6 Characterisation of the binary films

#### 2.6.1 Thickness

A digital micrometer with a sensitivity of 0.001 mm was used for the measurement of the thickness of the probiotic edible films. Thickness was calculated as the average of eight measurements taken from different regions of the film.

#### 2.6.2 Colour characteristics and opacity

Colour characteristics of the edible films were determined using a Hunterlab (Reston, USA) colourimeter as per (Fernández-Vázquez et al., 2014) with minor amendments. The CIELab colour scale was used to measure L* (black to white hue component), a* (red to green hue component) and b* (yellow to blue hue component) parameters (Zhang, Linforth, & Fisk, 2012). Opacity measurements were made according to the method described by Núñez-Flores et al., (2012). Film samples were cut into rectangles (0.7 × 1.5 cm^2^) and placed carefully on the surface of a plastic cuvette within the spectrophotometer cell after calibration with an air blank. The absorbance at 550 nm (A550) was measured using a UV-VIS spectrophotometer (Jenway Ltd., UK) and film opacity was calculated according to the formula:

(4) $$\text{Opacity}=\frac{{\text{A}}_{550}}{\text{thickness}}$$

#### 2.6.3 Tensile tests

Mechanical characterisation (tensile strength (TS) and elongation percentage (% E) at break) of the films was conducted using a TA-XT exponent texture analyser (Stable Micro Systems Ltd, Surrey, UK). Pre-conditioned edible films (54% RH, 25 °C for 3 days), cut in 20 × 80 mm rectangular shapes were placed between the tensile grips (A/TG) allowing a grip separation distance of 50 mm. For tensile tests, a 5 kg load cell was used with a cross-head speed of 1 mm/s. The following properties were calculated from the stress–deformation curves:

(5) $$\text{TS}=\frac{{\text{F}}_{\max}}{\text{A}}$$

(6) $$\%\;\mathrm{Ε=100}\times \frac{\text{L}}{{\text{L}}_{\text{0}}}$$

where: F_max_ = the force at break (N), A = the film thickness (μm), L = the film length at break (mm), L_0_ = the initial film length (mm).

#### 2.6.4 Water vapour permeability

Water vapour permeability (WVP) of the probiotic edible films was determined gravimetrically according to the method described by Galus and Lenart (2013) with minor modifications. Very briefly, samples were placed between two rubber rings on the top of glass cells containing silica gel (0% RH). The glass cells were transferred to a ventilated chamber maintained at 100% RH (pure water) and 25 °C. Weight increase of the glass cells containing silica gel was recorded over a 72 h time period. WVP was calculated according to the formula:

$$\text{WVP}=\frac{\Delta \text{m}\cdot \text{e}}{\text{A}\cdot \Delta \text{t}\cdot \Delta \text{p}}$$

where: Δm/Δt = the moisture uptake rate (g/s) from silica gel, A = the film area exposed to moisture transfer, e = the film thickness, and Δp = the water vapour pressure difference between the two sides of the film.

#### 2.6.5 Morphological characterisation using scanning electron microscopy

A small film specimen was carefully deposited onto carbon tabs (Agar Scientific, Stansted, UK) and coated with carbon (Agar turbo carbon coater) to improve conductivity. The scanning electron microscope analysis (SEM) was performed on a FEI Quanta 3D 200 dual beam Focused Ion Beam Scanning Electron Microscope (FIB-SEM). The images were acquired using secondary electron imaging at an accelerating voltage of 5–15 kV.

#### 2.6.6 Differential scanning calorimeter (DSC)

A power-compensated Perkin Elmer DSC-7 (Perkin Elmer Ltd., Beaconsfield, UK) was used for the measurement of the glass transition temperature of the edible films, as per Yonekura, Sun, Soukoulis, and Fisk (2014) with some amendments. A small amount of plasticised pre-weighed edible film (6–10 mg) was placed in a high-pressure, stainless steel pan and subjected to the following cooling – heating protocol: 1) cool from 25 to −120  °C at 50 °C min^−1^, 2) hold isothermally at −120 °C for 10 min, 3) heat from −120 to 200  °C at 5 °C min^−1^ and 4) cool from 200 to −120  °C at 50 °C min^−1^ 5) hold isothermally at −120 °C for 10 min, 6) heat from −120 to 200  °C at 5 °C min^−1^ and 7) cool from 200 to 25  °C at 50 °C min^−1^. The onset (T_g,on_) and midpoint glass transition temperatures (T_g,mid_) were calculated from the second heating step.

#### 2.6.7 Dynamic mechanical analysis (DMA)

The dynamic mechanical measurements were carried out using a Perkin Elmer DMA 8000 (Perkin Elmer Ltd., Beaconsfield, UK) operating in tension mode. The film samples were cut in 5 mm by 20 mm strips and conditioned at 54 ± 1% RH and 25 ± 1 °C for 72 h before analysis. The film samples were gripped in the tension geometry attachment and subject to static tension whilst measuring in oscillatory mode at frequencies of 0.1, 1 and 10 Hz Thermal sweeps were conducted by heating the samples at 3 °C min^−1^ between −80 and 180 °C (Martins et al., 2012). The storage modulus (E′), loss modulus (E″) and tanδ (E″/E′) were calculated at a frequency of 1 Hz with the glass transition temperature (T_g_) being defined as the peak value of tanδ. All analyses were carried out in duplicate.

### 2.7 Statistical analysis

Two-way ANOVA followed by Duncan's post hoc means comparison (p < 0.05) test was performed to evaluate the main effects of the investigated factors (starch and protein source type) on microbiological, physicochemical and mechanical data. Repeated measures ANOVA was used to identify the impact of storage time on the survival of L. rhamnosus GG. Principal component analysis (PCA) was performed to describe the interrelationships of film compositional profile and their respective microbiological, physicochemical and mechanical properties. All statistical treatments were performed using the MINITAB release 16 statistical software (Minitab Inc., PA, USA).

## 3 Results and discussion

### 3.1 Survival of *L. rhamnosus* GG during the drying process

**Fig. 1:**
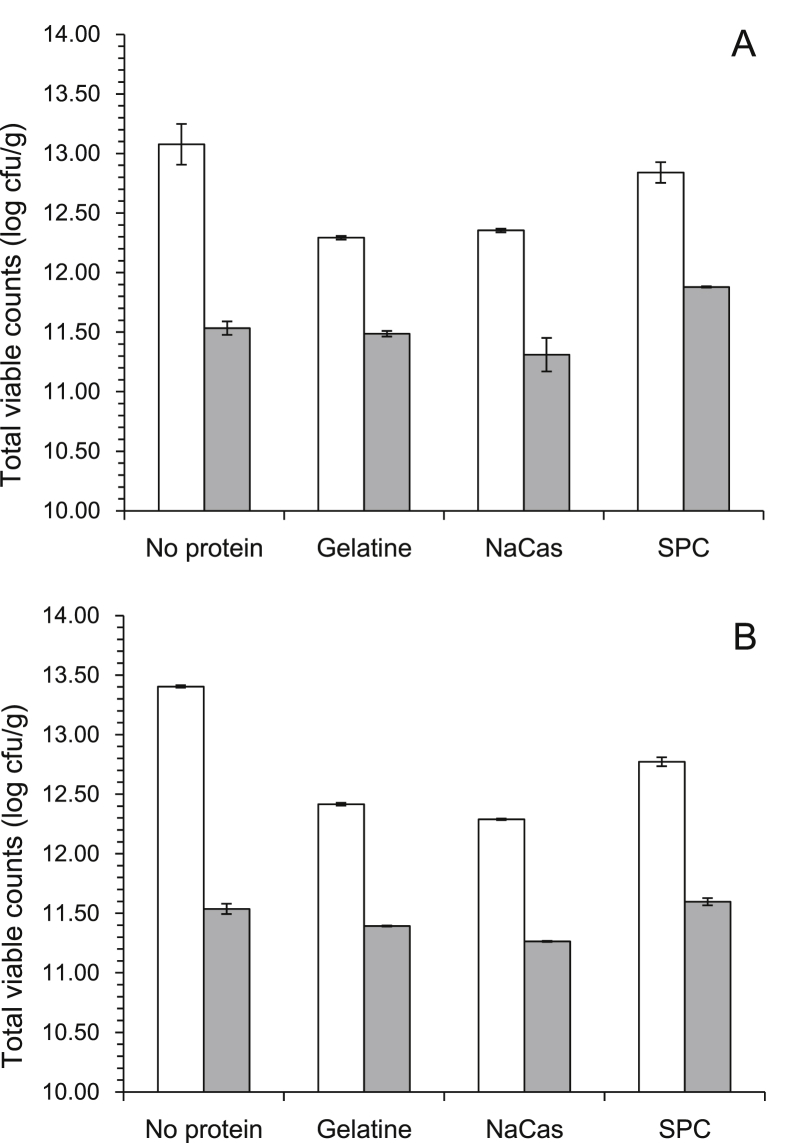
*L. rhamnosus GG* total viable counts during air drying (37 °C, 15 h) for each matrix composition (a = corn starch and b = rice starch based, white bar = start of drying, gray bar = end of drying).

The changes in total viable count (TVCs) of *L. rhamnosus* GG during the drying process are displayed in Fig. 1. Due to the physical state (liquid to gel-sol) transitions and changes in moisture content that occur during drying TVCs have been expressed on a total solids dry basis. In all cases, air drying was accompanied by a significant (p < 0.001) decrease of TVCs of *L. rhamnosus* GG ranging from 0.81 to 1.87 log CFU/g. According to ANOVA results, starch type had no significant impact (p > 0.05) on the inactivation of *L. rhamnosus* GG during air drying. A mean reduction of 1.15 and 1.21 log CFU/g was detected in corn and rice starch based systems respectively. A loss of 0.91, 1.03 and 1.07 log CFU/g was observed in the systems containing gelatine, sodium caseinate (NaCas) and SPC respectively, which is significantly lower (p < 0.01) than the losses detected in systems without protein (1.71 log CFU/g).

### 3.2 Inactivation kinetics of *L. rhamnosus* GG during storage

**Fig. 2:**
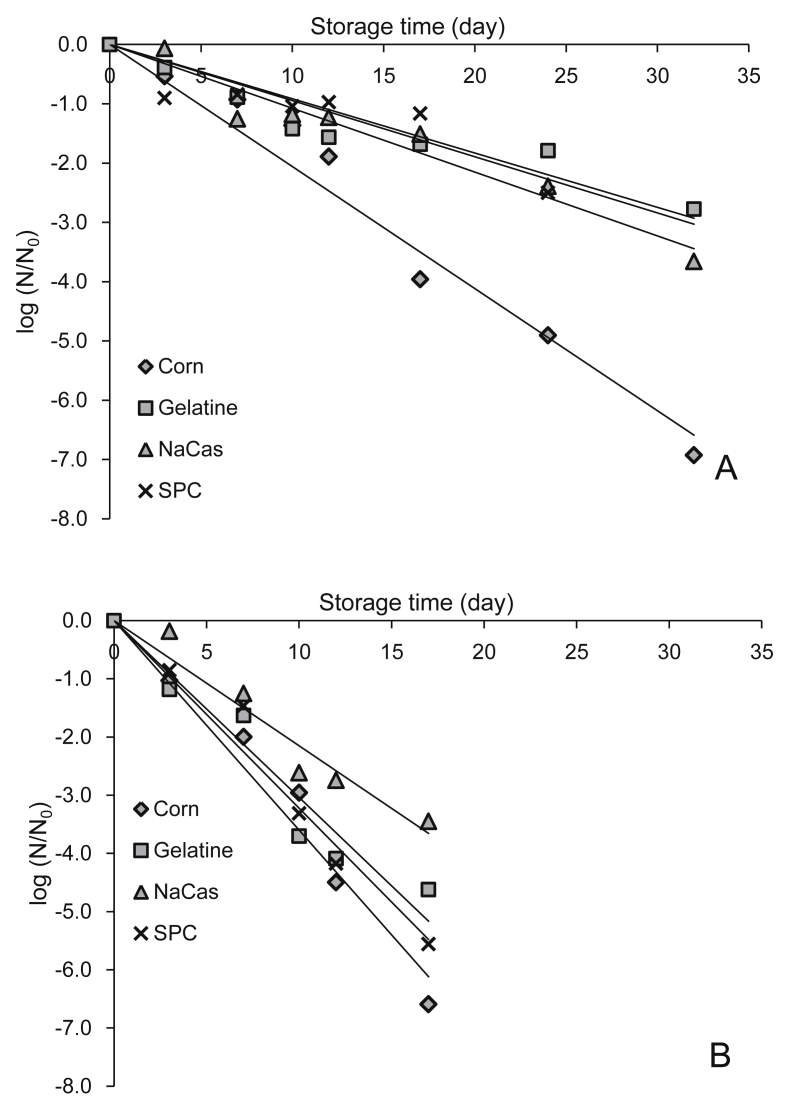
Effect of protein type (gelatine, sodium caseinate and soy protein concentrate) and storage temperature (A = 4 °C, B = 25 °C) on the inactivation of *L. rhamnosus GG* embedded in corn starch based edible films.

**Fig. 3:**
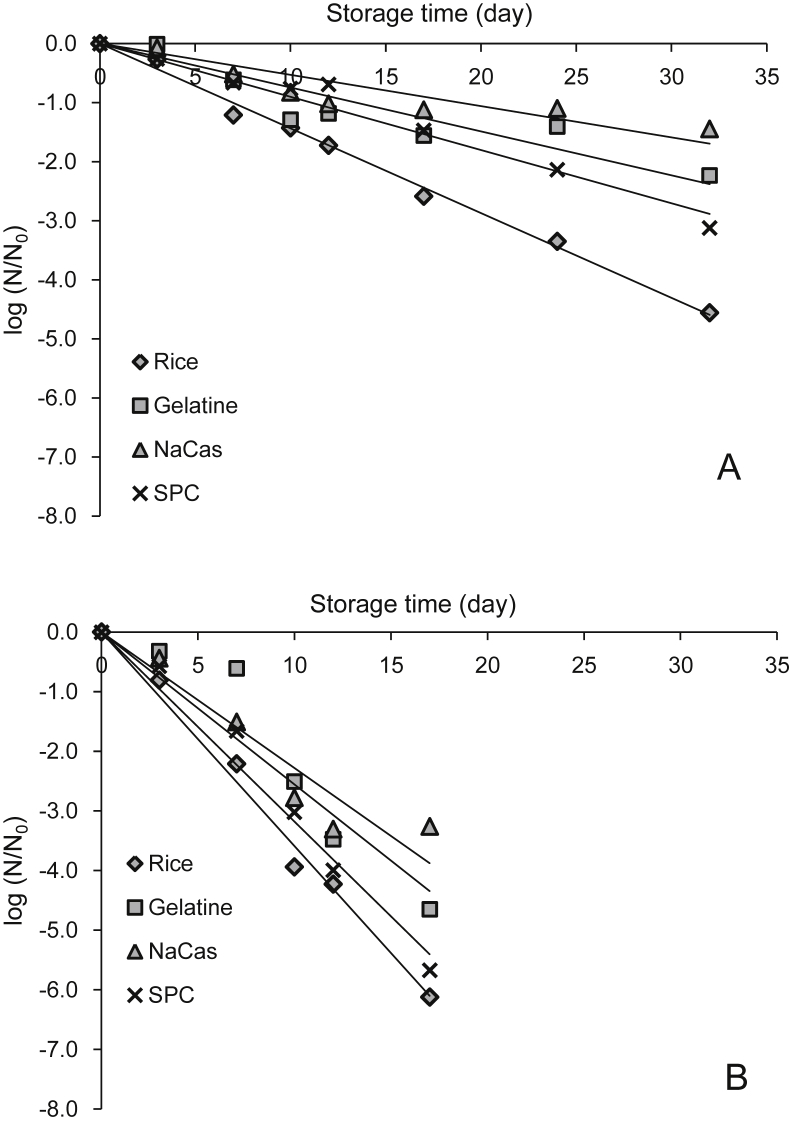
Effect of protein type (gelatine, sodium caseinate and soy protein concentrate) and storage temperature (A = 4 °C B = 25 °C) on the inactivation of *L. rhamnosus GG* embedded in rice starch based edible films.

The inactivation curves of *L. rhamnosus* GG immobilised in corn and rice starch based edible films are shown in Figs. 2 and 3 respectively. In all cases, inactivation of *L. rhamnosus* GG upon storage followed first order kinetics, inactivation rates are detailed in Table 1. At 4 °C films without protein exerted significantly (p < 0.001) higher inactivation rates. Rice starch based matrices enhanced the storage stability of *L. rhamnosus* GG (0.091 log CFU/day) compared to corn based systems (0.125 log CFU/day) at 4 °C, but no significant differences were detected in the stability of *L. rhamnosus* GG in the systems stored at room temperature (0.290 and 0.300 log CFU/day for rice and corn based films). In terms of protein addition, in general NaCas offered enhanced viability (p < 0.01) when compared to gelatin and SPC based films. Specifically in corn starch films, the ability of protein to enhance *L. rhamnosus* GG viability was found to be starch- and temperature-dependent, with protein type having a significant (p < 0.05) effect at room temperature. Whereas in rice starch films, proteins acted independently of storage temperature, according to the following order: NaCas < gelatine < SPC.

### 3.3 Probiotic film characterisation

#### 3.3.1 Morphological characterisation

**Fig. 4:**
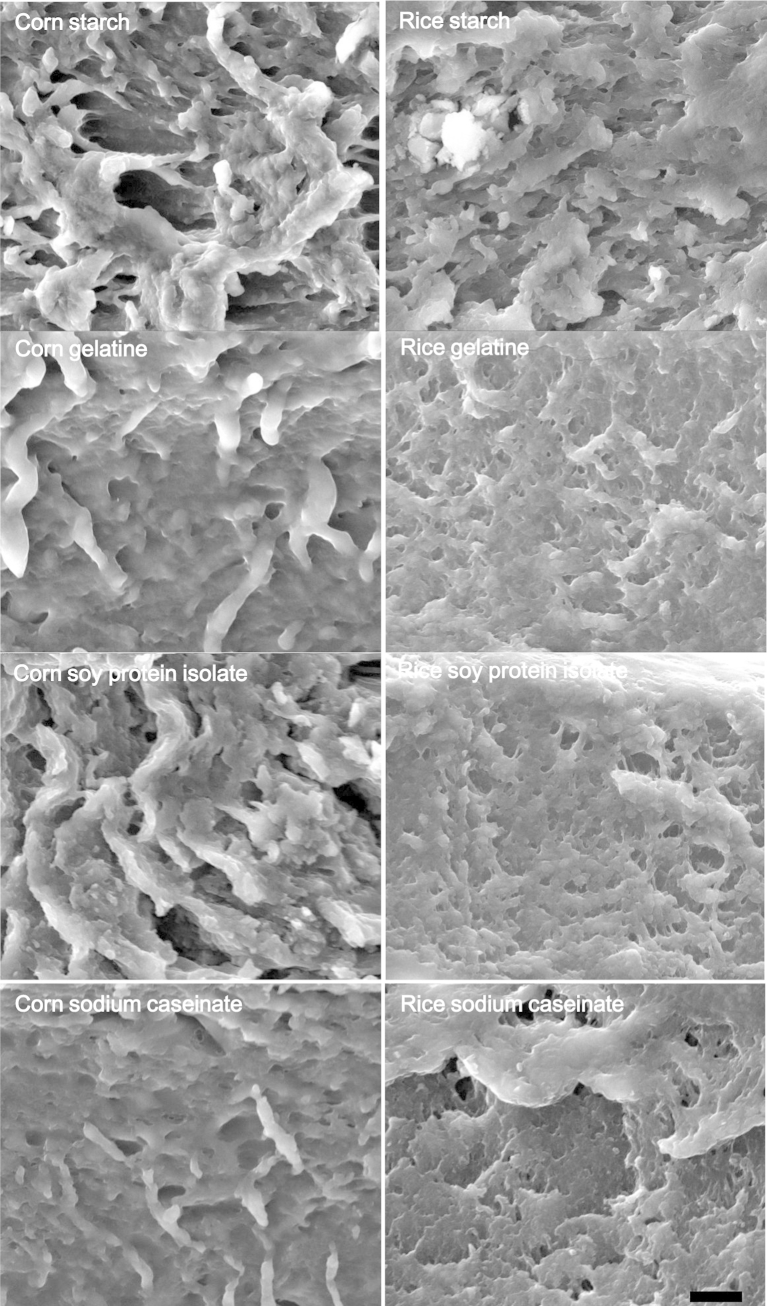
Cross-section of the starch-protein based edible films using Scanning Electron Microscopy. Scale bar = 10 μm.

Scanning electron microscopy (SEM) was used to visualise the cross-section of the edible films, identify their structural features and evaluate the cross-sectional homogeneity (Fig. 4). According to Fig. 4, starch type was the governing factor for the development of the microstructural features; corn starch was associated with the formation of a reticular, honeycomb-like structure with bud-like protrusions whilst rice starch based films exhibited a coarser, flaky-like more compact structure. However it should be noted that in both cases, films were characterised by an irregular, non-homogeneous structure with inner voids which is generally a marker of thermodynamical incompatibility of the present biopolymers. (Galus, Mathieu, Lenart, & Debeaufort, 2012).

In their study, Liu et al., (2005) investigated the impact of amylose to amylopectin ratio on the structure forming ability of starch and reported that, depending on the amylose to amylopectin ratio, heterogeneous structures are created via intermolecular (association of amylose with amylopectin branches to form double helices) and supramolecular (amylose double helices bundled with amylopectin) interactions. In addition, the increase of crystallinity due to post-drying physical state transitions e.g. starch retrogradation during conditioning, may also lead to alteration of the microstructure of starch based films leading to the development of more brittle and coarse structures.

It is well-established that film structures characterised by low porosity and high cohesiveness/compactness are associated with improved barrier and mechanical strength properties (Lacroix, 2009). As can be seen in Fig. 4, the addition of protein to the rice based films was associated with the development of a more compact and cohesive structure, presumably due to the ability of proteins to either interact with starch molecules via hydrogen bonding or hydrophobic interactions (Elgadir et al., 2012) thereby reducing the interspaces within the starch matrix. The evidence for corn was less clear (Fig. 4). Furthermore, it should be pointed out that, regardless of the film composition, it was not possible to visualize the living probiotic cells using the FIB-SEM, which indicates effective physical entrapment in the biopolymer matrix (Soukoulis et al., 2014).

#### 3.3.2 Colour and optical properties

Colour and optical properties are important features of edible films as they can directly affect the consumers’ preference and product choice (García, Pinotti, Martino, & Zaritzky, 2009). According to ANOVA results, starch type (corn vs. rice) did not significantly (p > 0.05) affect the measured luminosity L* (89.84 and 90.08 respectively), and red to green hue component a* (−0.965 and −0.950 respectively), of probiotic edible films. On the other hand, rice starch based edible films were characterised by significantly lower opacity values (ANOVA mean values were 3.54 vs. 4.30 for rice and corn starch respectively) and b* values (7.79 vs. 10.17). Parameters such as the film thickness, the crystallinity and crystallites mean size, the plasticiser type and amount as well as the refractive index, structural conformation and compatibility of the film components are known to influence the opacity of edible films (Fakhouri et al., 2013; Liu et al., (2005); Villalobos, Chanona, Hernández, Gutiérrez, & Chiralt, 2005; Zhang & Han, 2010).

Protein addition was accompanied in most cases by a significant increase in the film's opacity, green (-a*) and yellow (b*) colour intensity components (Table 2). In the case of the SPC containing films, an approximate 2-fold increase of the opacity values was observed, which may be indicative of its reduced miscibility with starch although visually it appeared homogenous (Galus, Lenart, Voilley, & Debeaufort, 2013). Finally, It should also be noticed that the presence of bacterial cells tended to slightly increase the opacity of the edible films although the differences were not significant (p > 0.05, data not shown). This is in agreement with previous reports (Kanmani & Lim, 2013; Soukoulis et al., 2014b).

#### 3.3.3 Thickness, tensile and thermo-mechanical properties

Starch type did not significantly influence the thickness of the edible films when evaluated by ANOVA (0.099 and 0.106 mm for rice and corn starch respectively) although there was a difference in the starch only films, indicating similar film forming properties of both materials when in the presence of proteins. In addition, only SPC was found to significantly (p < 0.01) increase film thickness (0.137, 0.079, 0.093 and 0.100 for SPC, gelatine, NaCas and no protein systems respectively). In agreement with our findings, Galus and Lenart (2013) and Fakhouri et al., (2013) reported a significant increase in the thickness of binary starch – soy protein edible films compared to the systems based exclusively on soy protein, and only a minor effect of gelatine concentration on edible film thickness.

Edible films should possess adequate mechanical strength and extensibility to withstand the stresses experienced during food processing, packaging and storage (Falguera, Quintero, Jiménez, Muñoz, & Ibarz, 2011). Parameters such as the structural conformation of the film's major components and their interactions, the presence of structure imperfections (voids, fissures, cracks) and the amount and type of plasticising agents have been reported to influence the mechanical profile of edible films (Falguera et al., 2011; Lacroix, 2009). In the present work, the plasticiser content was kept constant at 30% w/w of biopolymer total solids which facilitated the development of flexible and extensible structures without imparting any tackiness or brittleness. Moreover, tensile tests confirmed (data not shown) that the presence of probiotic bacterial cells did not influence the mechanical properties of the films (p > 0.05); this is in agreement with the previous findings of Kanmani and Lim (2013) and Gialamas, Zinoviadou, Biliaderis, and Koutsoumanis (2010).

Regarding the tensile test results (Table 3), both starch addition (p < 0.05) and protein type (p < 0.01) impacted tensile strength (TS) and extensibility (% E) per loading weight of probiotic edible films. Films based on rice starch in general had a lower tensile strength at break and a lower or equal elongation at break as indicated by ANOVA mean values for TS (0.42 vs. 0.64) and % E (17.8 vs. 29.5) for the rice and corn starch systems respectively. Notwithstanding the small differences in the starch amylose/amylopectin composition, we hypothesize that the altered mechanical strength and elongation properties of rice films compared to the corn starch based ones is related to their higher compactness as shown by SEM (Fig. 4) and to their modified glass transition temperatures.

**Fig. 5:**
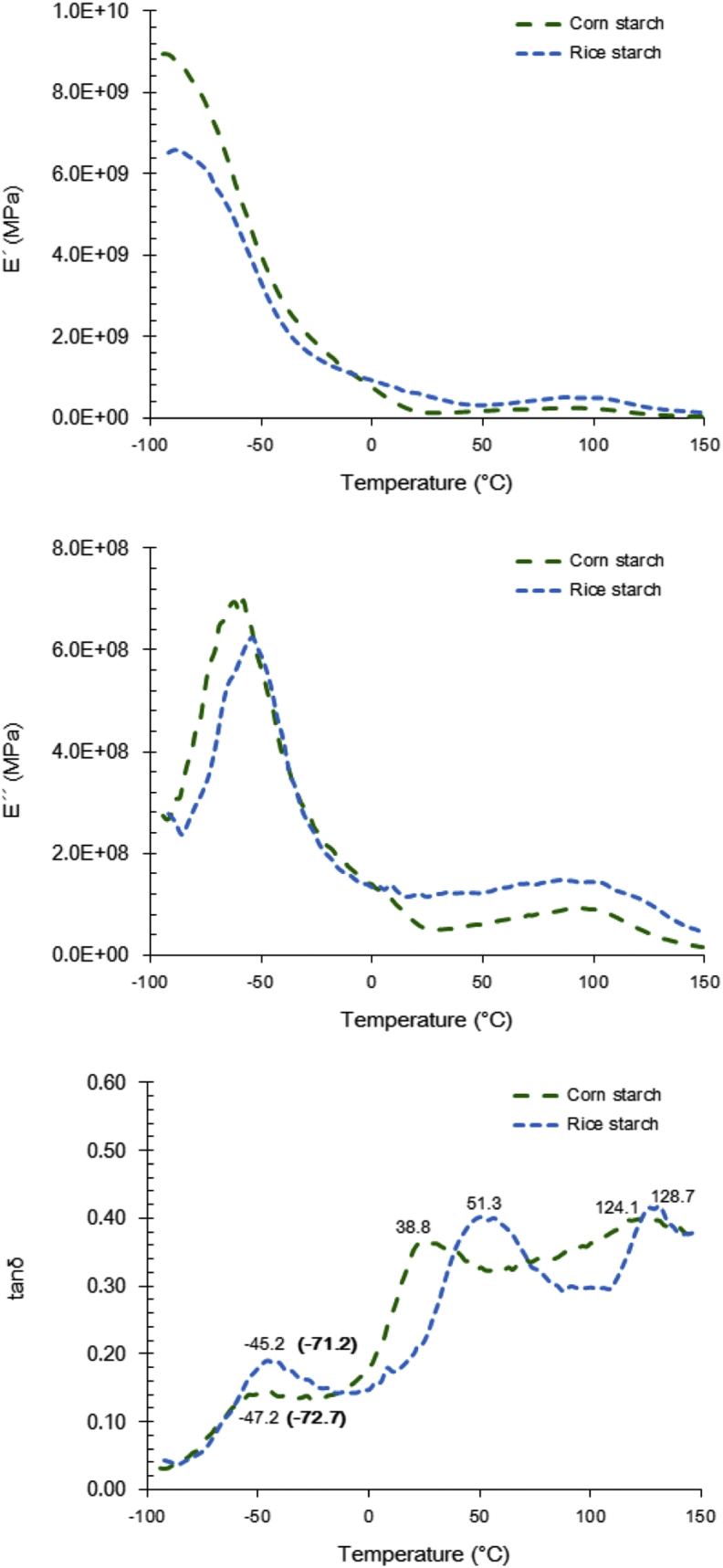
Dynamic mechanical analysis (DMA) of probiotic edible films containing corn or rice starch. Values marked in bold correspond to the midpoint glass transition temperature as determined using differential scanning calorimetry (DSC).

**Fig. 6:**
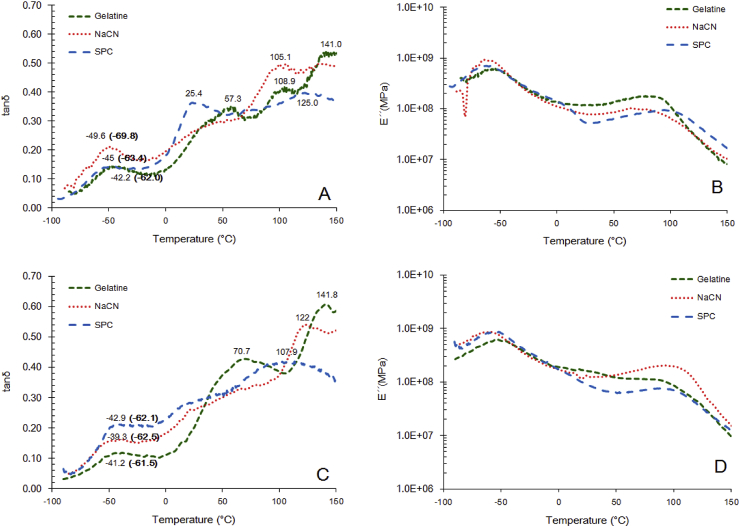
Dynamic mechanical analysis (DMA) of probiotic edible films comprised blends of protein and corn (A, B) or rice starch (C, D). Values marked in bold correspond to the midpoint glass transition temperature as determined using differential scanning calorimetry (DSC).

According to the DMA analysis (Figs. 5 and 6), two main physical state transitions for corn and rice starch systems were detected, indicating the occurrence of phase separation. The low temperature transition (−47.2 and −45.2 °C for corn and rice starch respectively) is possibly associated with a plasticiser (glycerol) rich region, whilst the higher temperature phase transition (38.8 and 51.3 °C) is indicative of the presence of a biopolymer rich regions (Ogale, Cunningham, Dawson, & Acton, 2000). The latter appears to be in accordance with the compositional aspects of the fabricated films, that is, the higher amylopectin to amylose ratio in the case of the rice starch. A similar behaviour was also attained in the case of gelatine – starch binary blends (57.3 vs. 70.7 °C for corn starch and rice starch respectively) whilst no remarkable differences were detected when sodium caseinate was used a protein source. In SPC-based systems, tanδ was peaked at 25.3 °C in the case of corn starch systems whilst rice starch containing films exerted a similar thermo-mechanical pattern to that of sodium caseinate. Finally, the physical state transitions detected at high temperatures (above 100 °C) can be attributed to the structural changes taking place due to water evaporation.

DSC analysis confirmed also the presence of the β-relaxation (Figs. 5 and 6, low temperatures highlighted in bold) peak whilst in all cases no α-relaxation in the region 0–150 °C was observed in agreement to previous studies (Denavi et al., 2009; Ogale et al., 2000). As a general rule, the systems fabricated with rice starch were characterised by higher T_g_ values compared to the corn starch analogues. It well established that plasticiser type and amount impact the thermophysical profile of starch based food systems (Al-Hassan & Norziah, 2012). However, in the present study, both plasticiser (25.3 vs. 25.1 g/100 g of film) and residual water content (15.72 vs. 16.19 H_2_O g/100 g of film) did not significantly vary across the tested systems. In this context, it is postulated that the elevated T_g_ values in the case of rice starch films can be attributed to their higher amylopectin content compared to the corn starch analogues (Janssen & Moscicki, 2009). In addition, the lower amylose content of rice starch based systems has been also proposed as elevating the T_g_ via a supramolecular cross-linkages promoting mechanism (Chung, Lee, & Lim, 2002). Incorporation of proteins in the probiotic films induced a significant increase in their glass transition temperature. However, T_g_ did not exert any specific dependence on protein source utilised for the preparation of the films. It is therefore assumed, that there is no difference in the ability of protein molecules to form linkages with the amorphous starch components via hydrogen bonding and/or hydrophobic interactions (Elgadir et al., 2012).

#### 3.3.4 Water vapour permeability

**Fig. 7:**
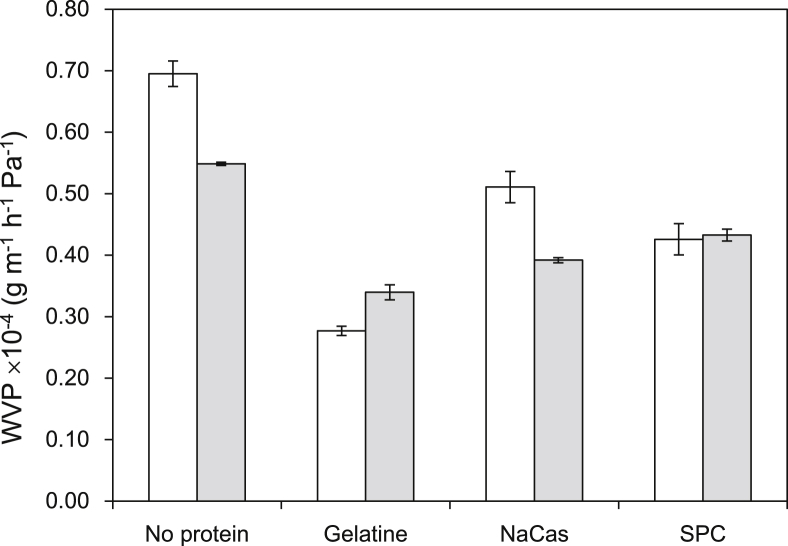
Water vapour permeability (WVP) of the probiotic edible films based on corn (white bars) or rice starch (gray bars).

Diffusivity of films to gases is generally influenced by several factors with composition, physical state (crystalline or amorphous), thickness, biopolymer structuring and intermolecular interactions, plasticiser type and content and storage conditions (relative humidity and temperature) being the most critical (Bertuzzi, Castro Vidaurre, Armada, & Gottifredi, 2007; Lacroix, 2009; McHugh, Aujard, & Krochta, 1994). Fabrication of edible films with low permeability to water vapour is generally required to effectively control shelf-life impairing reactions (e.g. lipid oxidation, vitamin reaction, browning), structural and textural collapse and microbial spoilage. Film water vapour permeability (Fig. 7) decreased significantly (p < 0.001) in the presence of proteins, with gelatine conferring the most prominent effect. Al-Hassan and Norziah (2012) reported that the presence of gelatine in sago starch films plasticised with glycerol resulted in a reduction of WVP due to its ability to interact with starch chain polymers via hydrogen bonding. Similarly, Chinma, Ariahu, and Abu (2012) demonstrated that the decreased WVP of cassava starch-SPC films is associated with the ability of proteins to interact with starch, reducing the hydrodynamic free volume between the biopolymers and thus hindering sterically the molecular mobility of water. In addition, the structuring properties of proteins leading to cross-linked/entangled networks have also been reported as another parameter that restricts water vapour transmission rates. The latter could be significant here, as the presence of protein was accompanied by the formation of more compact, less porous structures according to SEM analysis. In addition, the less hydrophilic character of SPC (Chinma et al., 2012) and NaCas (Arvanitoyannis, Psomiadou, & Nakayama, 1996) can also explain lower WVP. With regard to the films containing no protein, corn starch probiotic films exerted poor barrier properties compared to rice starch which is supported by SEM images showing a more porous network in the corn starch films (Fig. 4).

### 3.4 General discussion

Edible films due to their sustainable nature, appropriate physical and chemical properties and versatility in application are proposed as potential vehicles for the delivery of bioactive compounds (Falguera et al., 2011; López de Lacey et al., 2012). Moreover, they may provide a feasible and versatile carrier for the delivery of probiotics under extreme conditions during food processing such as baking (Soukoulis et al., 2014). In the present work, two sources of native starch were selected due to their good film forming ability (Kramer, 2009) whereas proteins were selected on the basis of their commercial availability and proposed benefit on probiotics viability. To date, data on the effect of starch type on probiotic strain viability during edible film formation is rather scarce. Kanmani and Lim (2013) reported a decrease of the viable counts of a symbiotic blend of Lactobacilli (*Lactobacillus reuteri*, *L. acidophilus* and *Lactobacillus plantarum*) in the presence of pure native starches (potato, tapioca and corn) compared to pure pullulan systems, although no clear effects of starch type on TVCs throughout drying were reported.

According to our findings, a 3- to 4-fold and 5- to 7-fold increase of the viability of *L. rhamnosus* GG was observed in the presence of proteins for corn and rice starch based films respectively. Gelatine and sodium caseinate were associated with the highest protective effect against osmotic and heat stress induced injuries during drying especially in the rice based films. It has been demonstrated that proteins can enhance probiotics survival by scavenging free radicals and supplying micronutrients (such as peptides and amino acids) essential for the growth of weakly proteolytic probiotic bacteria (Burgain, Gaiani, Cailliez-Grimal, Jeandel, & Scher, 2013; Burgain et al.,2013, 2014; Dave & Shah, 1998; Soukoulis et al., 2014). Due to the moderately low temperature implemented for the evaporation of the film forming solutions and drying process, it can be deduced that the observed effects on *L. rhamnosus* GG are primarily osmotically driven (Ghandi, Powell, Chen, & Adhikari, 2012).

Here we hypothesise that factors such as the bacteria's adaptability in the drying medium as well as their ability to adhere on the existing biopolymers played a crucial role in sustaining the viability of *L. rhamnosus* GG throughout drying. During the first 4–5 h of drying, water activity was higher than the threshold required for the growth of *Lactobacilli* (aw = 0.91) providing optimum conditions for the adaptation and growth of the living cells in the drying medium. In addition, the presence of proteins provided peptides and amino acids for the growth of the bacteria compared to pure starch solutions, enhancing their ability to withstand the sub-lethal effect of the increasing osmotic pressure due to the decline of water activity. On the other hand, it has been reported that the adhesion properties of probiotic cells can also reflect their ability to overcome acute lethal processes such as severe heating, osmolysis and physicochemical stress associated with processing and gastro-intestinal conditions (Burgain, Gaiani, Cailliez-Grimal, et al., 2013; Burgain et al., 2013). Probiotic and lactic acid bacteria exert the ability to interact with biopolymers such as polysaccharides and proteins via electrostatic or hydrophobic interactions or short-range forces e.g. van der Waals and hydrogen bonding (Deepika & Charalampopoulos, 2010). *L. rhamnosus* GG cells are predominantly negatively-charged over a broad pH range (3–10) whilst they are characterised by high surface hydrophobicity (Deepika, Green, Frazier, & Charalampopoulos, 2009). Thus, it should be expected that the adhesion of *Lactobacillus rhamnsosus* GG to the drying medium is governed mainly via hydrogen bonding or hydrophobic interactions. Finally, entrapment of the bacterial cells in the formed biopolymer networks (surpassing the critical concentration c* during the last stage of drying) and prevention of water loss from their cellular membranes (Fu & Chen, 2011) can also be considered as an additional factor shielding *L. rhamnosus* GG during drying.

Inactivation of probiotics during storage is mainly influenced by factors such as bacteria species/strain, storage temperature, residual water content, presence of protective carriers, oxidative stress and physical state transitions (Fu & Chen, 2011). Immobilisation of living cells in edible films is challenging as the presence of plasticisers increases the molecular mobility of water, accelerating lethal enzymatic and chemical reactions e.g. lipid peroxidation of cytoplasmic membranes. In addition, the high permeability of films to gases e.g. water vapour and oxygen can also impact adversely the viability of bacterial cells. To the best of our knowledge, matrix composition (polysaccharides and protein type, presence of prebiotics, type and amount of plasticiser) and storage temperature possess a dominant role on storage stability of *L. rhamnosus* GG (Kanmani & Lim, 2013; López de Lacey et al., 2012; Soukoulis et al., 2014). In the present work, it has been confirmed that low temperature storage conditions (fridge) and protein addition prolonged shelf-life (herein defined as the time required to reaching a minimum of 6 log CFU/g) which ranged from 27 to 96 days. It was also observed that the use of rice starch enhanced the viability of *L. rhamnosus* GG, particularly at 4 °C. It should also be pointed out that the shelf life of starch based films at 25 °C (up to 24 days) is of relevance to short shelf life foodstuffs such as bakery products.

According to DMA and DSC analysis (Figs. 5 and 6), it was found that T_storage_ >> T_g_ suggesting that all matrices were in the rubbery state and thus, the inactivation kinetics of *L. rhamnosus* GG during storage cannot be phenomena associated with the solutes’ sterical hindrance as in the case of anhydrobiotics (Soukoulis, Behboudi-Jobbehdar, Yonekura, Parmenter, & Fisk, 2014a). However, the elevation of T_g_ in the case of protein addition could be considered as a secondary factor explaining the inactivation rate reduction observed in the specific systems.

**Fig. 8:**
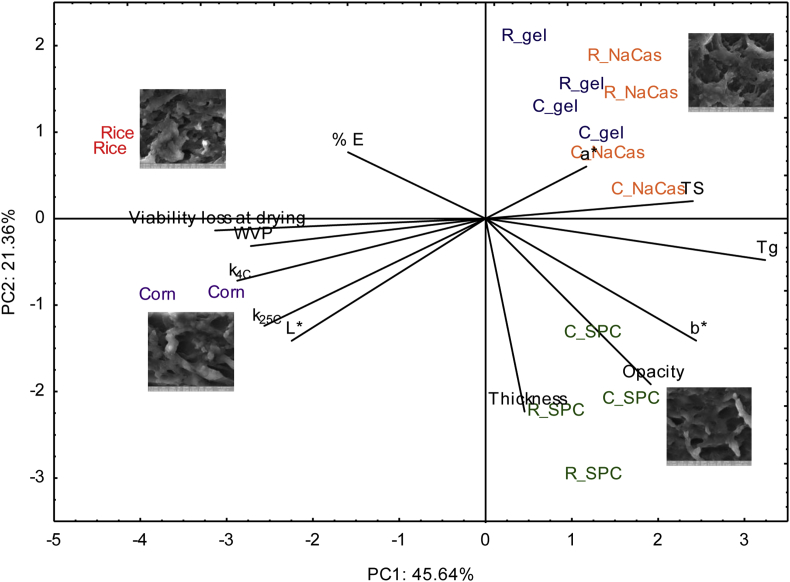
Principal components analysis (PCA) based on the microbiological, physicochemical and mechanical properties of probiotic edible films comprised of different type of starch (corn and rice) and proteins (gelatine, sodium caseinate and soy protein concentrate), replicates are shown.

Physical, thermo-mechanical and microbiological data was subjected to PCA analysis, this is presented in Fig. 8 with PC1 and PC2 explaining 45% and 21% of the variance. PCA analysis resolved the film systems by protein inclusion (PC1) and by protein type (PC2). The main variables separating the data were the inactivation rate during storage and T_g_ (PC1) and film properties (PC2). In general, inactivation rates of *L. rhamnosus* GG (k_4C_ and k_25C_) was inversely correlated with T_g_ of the films.

Protein incorporation into the film enhanced the storage stability of *L. rhamnosus* GG with an improvement of *L. rhamnosus* GG survival rates ranging from 10.6 to 40% and 11.1–36.3% (at 25 °C) as well as from 47.5 to 55% and 36.8–62.5% (at 5 °C) shown in the corn and rice starch based systems respectively. It is therefore assumed that parameters such as the enhanced adhesion properties of *L. rhamnosus* GG and hindering of solute molecular mobility via the formation of intermolecular linkages between proteins and starch, may further explain the beneficial action of proteins (primarily gelatine and sodium caseinate) in promoting *L. rhamnosus* GG storage stability.

Apart from the physical state, the structural conformation of the films (biopolymers entanglement, matrix compactness and porosity) influences the exposure level of the bacteria to the toxic external environmental condition. Recently, we have demonstrated that the poor coverage of *L. rhamnosus* GG in sodium alginate coated bread crust samples was responsible for its higher lethality compared to the sodium alginate/whey protein concentrate systems (Soukoulis et al., 2014). According to Fig. 8, inactivation of *L. rhamnosus* GG was positively associated with WVP and negatively associated with T_g_ suggesting that a suppressed permeability of film structures to gases (hereby only for water vapour) is generally associated with increased survival rates. The latter is of particular importance as high WVP rates increase the plasticising effect of solutes and consequently raise the lethal biochemical reaction rates. Finally, it should be stated that a positive correlation between the loss percentage of *L. rhamnosus* GG throughout drying and inactivation rates during storage was obtained, which implies that osmotically injured cells during the dehydration process exert a poorer ability to compete in the hostile ambient storage conditions.

In conclusion, in the present study it was shown that the immobilisation of *L. rhamnosus* GG in plasticised starch based matrices is a viable strategy to deliver probiotics into food products. Whilst edible films do not allow long term storage of probiotics due to their physical state (rubbery, high plasticiser inclusion), they provide a good medium for intermediate moisture short shelf-life foods. Edible films based on binary starch-gelatine or starch-sodium caseinate blends exerted the best *L. rhamnosus* GG survival without compromising mechanical, optical and barrier properties (Fig. 8) and the most compact (SEM) lowest VWP films as shown in the rice exemplar were most stable over shelf life. In continuation to our previous studies, we have demonstrated that probiotic efficacy in functional foods with elevated plasticiser content can be achieved by controlling/optimising the physicochemical and structural properties of the edible films.

## Figures and Tables

**Table 1:** Inactivation rates of *L. rhamnosus GG* embedded in plasticised starch-protein matrices stored at 4 and 25 °C.

Matrix type	Inactivation rate at 4 °C (R^2^) k_4_ (log CFU/g day^−1^)	Shelf-life‡ at 4 °C (days)	Inactivation rate at 25 °C (R^2^) k_25_ (log CFU/g day^−1^)	Shelf-life at 25 °C (days)
Corn starch	0.206^e^ (0.966)	27	0.360^e^ (0.968)	16
Corn/Gelatine	0.092^c^ (0.859)	59	0.304^c^ (0.928)	18
Corn/Sodium caseinate	0.108^c^ (0.948)	48	0.215^a^ (0.946)	24
Corn/SPC	0.095^c^ (0.812)	61	0.322^d^ (0.968)	18
Rice starch	0.144^d^ (0.994)	38	0.358^e^ (0.989)	15
Rice/Gelatine	0.074^b^ (0.837)	72	0.256^b^ (0.898)	21
Rice/Sodium caseinate	0.054^a^ (0.883)	96	0.228^a^ (0.902)	23
Rice/SPC	0.091^c^ (0.965)	61	0.318^cd^ (0.974)	17

^a–e^ Different letter between the rows indicate significant difference (p < 0.05) according to Duncan's means post hoc comparison test.

‡ Refers to the time (in days) required the viable bacteria counts to decline at the value of 6 log cfu/g.

**Table 2:** Colour characteristics and opacity of starch-protein based edible films containing *L. rhamnosus GG*.

Matrix type	L*	a*	b*	Opacity
Corn starch	90.70 ± 0.02^bc^	−1.21 ± 0.08^a^	7.93 ± 0.02^b^	2.77 ± 0.04^bc^
Corn/Gelatine	88.82 ± 0.41^a^	−1.06 ± 0.01^a^	10.32 ± 0.50^c^	4.63 ± 0.18^d^
Corn/Sodium caseinate	89.60 ± 0.15^ab^	−0.42 ± 0.23^c^	11.94 ± 1.54^cd^	3.61 ± 0.13^c^
Corn/SPC	90.27 ± 0.79^abc^	−1.17 ± 0.04^a^	10.49 ± 0.50^c^	6.20 ± 0.29^e^
Rice starch	92.11 ± 0.16^c^	−1.01 ± 0.15^b^	2.89 ± 0.48^a^	1.73 ± 0.11^a^
Rice/Gelatine	88.29 ± 0.31^a^	−1.36 ± 0.02^a^	7.38 ± 0.52^b^	2.06 ± 0.06^ab^
Rice/Sodium caseinate	88.94 ± 0.23^a^	−0.36 ± 0.19^c^	7.46 ± 0.52^b^	3.30 ± 0.43^c^
Rice/SPC	90.99 ± 0.20^bc^	−1.07 ± 0.10^ab^	13.51 ± 0.58^d^	7.05 ± 0.41^f^

^a–f^ Different letter between the rows indicate significant difference (p < 0.05) according to Duncan's means post hoc comparison test.

**Table 3:** Mechanical characterisation of the starch-protein based edible films containing *L. rhamnosus GG*.

Matrix type	Thickness (mm)	Tensile strength at break TS (MPa)	Elongation at break E (%)
Corn starch	0.131 ± 0.001^b^	2.84 ± 0.21^a^	48.2 ± 6.6^c^
Corn/Gelatine	0.072 ± 0.003^a^	7.92 ± 0.70^c^	52.8 ± 4.4^c^
Corn/Sodium caseinate	0.091 ± 0.001^a^	5.68 ± 0.61^b^	11.3 ± 0.9^a^
Corn/SPC	0.137 ± 0.005^b^	9.10 ± 0.89^c^	5.7 ± 0.2^a^
Rice starch	0.069 ± 0.001^a^	2.26 ± 0.16^a^	26.4 ± 2.1^b^
Rice/Gelatine	0.086 ± 0.009^a^	6.10 ± 0.53^b^	22.3 ± 2.9^b^
Rice/Sodium caseinate	0.089 ± 0.001^a^	5.25 ± 0.48^b^	16.4 ± 1.9^ab^
Rice/SPC	0.137 ± 0.008^b^	7.08 ± 0.31^c^	6.2 ± 0.6^a^

^a–c^ Different letter between the rows indicate significant difference (p < 0.05) according to Duncan's means post hoc comparison test.

## References

[bib1] Al-Hassan A.A., Norziah M.H. (2012). Starch–gelatin edible films: water vapor permeability and mechanical properties as affected by plasticizers. Food Hydrocolloids.

[bib2] Altamirano-Fortoul R., Moreno-Terrazas R., Quezada-Gallo A., Rosell C.M. (2012). Viability of some probiotic coatings in bread and its effect on the crust mechanical properties. Food Hydrocolloids.

[bib3] Arvanitoyannis I., Psomiadou E., Nakayama A. (1996). Edible films made from sodium casemate, starches, sugars or glycerol. Part 1. Carbohydrate Polymers.

[bib4] Behboudi-Jobbehdar S., Soukoulis C., Yonekura L., Fisk I. (2013). Optimization of spray-drying process conditions for the production of maximally viable microencapsulated L. acidophilus NCIMB 701748. Drying Technology.

[bib5] Bertuzzi M.A., Castro Vidaurre E.F., Armada M., Gottifredi J.C. (2007). Water vapor permeability of edible starch based films. Journal of Food Engineering.

[bib6] Burgain J., Gaiani C., Cailliez-Grimal C., Jeandel C., Scher J. (2013). Encapsulation of Lactobacillus rhamnosus GG in microparticles: influence of casein to whey protein ratio on bacterial survival during digestion. Innovative Food Science & Emerging Technologies.

[bib7] Burgain J., Gaiani C., Francius G., Revol-Junelles A.M., Cailliez-Grimal C., Lebeer S. (2013). In vitro interactions between probiotic bacteria and milk proteins probed by atomic force microscopy. Colloids and Surfaces B: Biointerfaces.

[bib8] Burgain J., Gaiani C., Linder M., Scher J. (2011). Encapsulation of probiotic living cells: from laboratory scale to industrial applications. Journal of Food Engineering.

[bib9] Burgain J., Scher J., Lebeer S., Vanderleyden J., Cailliez-Grimal C., Corgneau M. (2014). Significance of bacterial surface molecules interactions with milk proteins to enhance microencapsulation of Lactobacillus rhamnosus GG. Food Hydrocolloids.

[bib10] Champagne C.P., Ross R.P., Saarela M., Hansen K.F., Charalampopoulos D. (2011). Recommendations for the viability assessment of probiotics as concentrated cultures and in food matrices. International Journal of Food Microbiology.

[bib11] Chinma C.E., Ariahu C.C., Abu J.O. (2012). Development and characterization of cassava starch and soy protein concentrate based edible films. International Journal of Food Science & Technology.

[bib12] Chung H.-J., Lee E.-J., Lim S.-T. (2002). Comparison in glass transition and enthalpy relaxation between native and gelatinized rice starches. Carbohydrate Polymers.

[bib13] Cook M.T., Tzortzis G., Charalampopoulos D., Khutoryanskiy V.V. (2012). Microencapsulation of probiotics for gastrointestinal delivery. Journal of Controlled Release.

[bib14] Dave R.I., Shah N.P. (1998). Ingredient supplementation effects on viability of probiotic bacteria in Yogurt. Journal of Dairy Science.

[bib15] Deepika G., Charalampopoulos D., Laskin A.I., Sariaslani S., Gadd G.M. (2010). Chapter 4-Surface and adhesion properties of lactobacilli. http://www.sciencedirect.com/science/article/pii/S0065216410700046.

[bib16] Deepika G., Green R.J., Frazier R.A., Charalampopoulos D. (2009). Effect of growth time on the surface and adhesion properties of Lactobacillus rhamnosus GG. Journal of Applied Microbiology.

[bib17] Denavi G., Tapia-Blácido D.R., Añón M.C., Sobral P.J.A., Mauri A.N., Menegalli F.C. (2009). Effects of drying conditions on some physical properties of soy protein films. Journal of Food Engineering.

[bib18] Elgadir M.A., Akanda M.J.H., Ferdosh S., Mehrnoush A., Karim A.A., Noda T. (2012). Mixed biopolymer systems based on starch. Molecules.

[bib19] Fakhouri F.M., Costa D., Yamashita F., Martelli S.M., Jesus R.C., Alganer K. (2013). Comparative study of processing methods for starch/gelatin films. Carbohydrate Polymers.

[bib20] Falguera V., Quintero J.P., Jiménez A., Muñoz J.A., Ibarz A. (2011). Edible films and coatings: structures, active functions and trends in their use. Trends in Food Science & Technology.

[bib21] FAO/WHO. (2002). http://www.who.int/foodsafety/publications/fs_management/en/probiotics.pdf.

[bib22] FAO/WHO (2011). Milk and milk products. ftp://ftp.fao.org/codex/publications/booklets/milk/Milk_2011_EN.pdf.

[bib51] Fernández-Vázquez R., Hewson L., Fisk I.D., Hernanz Vila D., Mira F., Vicario I.M. (2014). Colour influences sensory perception and liking of orange juice. Flavour.

[bib23] Fu N., Chen X.D. (2011). Towards a maximal cell survival in convective thermal drying processes. Food Research International.

[bib24] Galus S., Lenart A. (2013). Development and characterization of composite edible films based on sodium alginate and pectin. Journal of Food Engineering.

[bib25] Galus S., Lenart A., Voilley A., Debeaufort F. (2013). Effect of oxidized potato starch on the physicochemical properties of soy protein isolate-based edible films. Food Technology and Biotechnology.

[bib26] Galus S., Mathieu H., Lenart A., Debeaufort F. (2012). Effect of modified starch or maltodextrin incorporation on the barrier and mechanical properties, moisture sensitivity and appearance of soy protein isolate-based edible films. Innovative Food Science & Emerging Technologies.

[bib27] García M., Pinotti A., Martino M., Zaritzky N., Huber K.C., Embuscado M.E. (2009). Characterization of starch and composite edible films and coatings. Edible films and coatings for food applications.

[bib28] Ghandi A., Powell I., Chen X.D., Adhikari B. (2012). Drying kinetics and survival studies of dairy fermentation bacteria in convective air drying environment using single droplet drying. Journal of Food Engineering.

[bib29] Gialamas H., Zinoviadou K.G., Biliaderis C.G., Koutsoumanis K.P. (2010). Development of a novel bioactive packaging based on the incorporation of Lactobacillus sakei into sodium-caseinate films for controlling Listeria monocytogenes in foods. Food Research International.

[bib30] Jankovic I., Sybesma W., Phothirath P., Ananta E., Mercenier A. (2010). Application of probiotics in food products-challenges and new approaches. Current Opinion in Biotechnology.

[bib31] Janssen L., Moscicki L. (2009). Thermoplastic starch.

[bib32] Kanmani P., Lim S.T. (2013). Development and characterization of novel probiotic-residing pullulan/starch edible films. Food Chemistry.

[bib33] Kourkoutas Y., Bekatorou A., Banat I.M., Marchant R., Koutinas A.A. (2004). Immobilization technologies and support materials suitable in alcohol beverages production: a review. Food Microbiology.

[bib34] Kramer M., Huber K.C., Embuscado M.E. (2009). Structure and function of starch-based edible films and coatings. Edible films and coatings for food applications.

[bib35] Lacroix M., Huber K.C., Embuscado M.E. (2009). Mechanical and permeability properties of edible films and coatings for food and pharmaceutical applications. Edible films and coatings for food applications.

[bib36] Liu Z.,H., Han J.H. (2005). Film-forming characteristics of starches. Journal of Food Science.

[bib37] López de Lacey A.M., López-Caballero M.E., Gómez-Estaca J., Gómez-Guillén M.C., Montero P. (2012). Functionality of Lactobacillus acidophilus and Bifidobacterium bifidum incorporated to edible coatings and films. Innovative Food Science & Emerging Technologies.

[bib38] López de Lacey A.M., López-Caballero M.E., Montero P. (2014). Agar films containing green tea extract and probiotic bacteria for extending fish shelf-life. LWT Food Science and Technology.

[bib39] Martins J.T., Cerqueira M.A., Bourbon A.I., Pinheiro A.C., Souza B.W.S., Vicente A.A. (2012). Synergistic effects between κ-carrageenan and locust bean gum on physicochemical properties of edible films made thereof. Food Hydrocolloids.

[bib40] McHugh T.H., Aujard J.F., Krochta J.M. (1994). Plasticized whey protein edible films: water vapor permeability properties. Journal of Food Science.

[bib41] Núñez-Flores R., Giménez B., Fernández-Mart¡n F., López-Caballero M.E., Montero M.P., Gómez-Guillén M.C. (2012). Role of lignosulphonate in properties of fish gelatin films. Food Hydrocolloids.

[bib42] Ogale A.A., Cunningham P., Dawson P. l., Acton J.C. (2000). Viscoelastic, Thermal, and microstructural characterization of soy protein isolate films. Journal of Food Science.

[bib43] Saad N., Delattre C., Urdaci M., Schmitter J.M., Bressollier P. (2013). An overview of the last advances in probiotic and prebiotic field. LWT Food Science and Technology.

[bib44] Soukoulis C., Behboudi-Jobbehdar S., Yonekura L., Parmenter C., Fisk I. (2014). Impact of milk protein type on the viability and storage stability of Microencapsulated Lactobacillus acidophilus NCIMB 701748 using Spray drying. Food and Bioprocess Technology.

[bib45] Soukoulis C., Behboudi-Jobbehdar S., Yonekura L., Parmenter C., Fisk I.D. (2014). Stability of Lactobacillus rhamnosus GG in prebiotic edible films. Food Chemistry.

[bib46] Soukoulis C., Yonekura L., Gan H.-H., Behboudi-Jobbehdar S., Parmenter C., Fisk I. (2014). Probiotic edible films as a new strategy for developing functional bakery products: the case of pan bread. Food Hydrocolloids.

[bib47] Villalobos R., Chanona J., Hernández P., Gutiérrez G., Chiralt A. (2005). Gloss and transparency of hydroxypropyl methylcellulose films containing surfactants as affected by their microstructure. Food Hydrocolloids.

[bib48] Yonekura L., Sun H., Soukoulis C., Fisk I. (2014). Microencapsulation of Lactobacillus acidophilus NCIMB 701748 in matrices containing soluble fibre by spray drying: technological characterization, storage stability and survival after in vitro digestion. Journal of Functional Foods.

[bib49] Zhang Y., Han J. h (2010). Crystallization of high-amylose starch by the addition of plasticizers at low and intermediate concentrations. Journal of Food Science.

[bib50] Zhang C., Linforth R., Fisk I.D. (2012). Cafestol extraction yield from different coffee brew mechanisms. Food Research International.

